# Effect of tempol and straw size on rooster sperm quality and fertility after post-thawing

**DOI:** 10.1038/s41598-022-16507-6

**Published:** 2022-07-16

**Authors:** Abouzar Najafi, Mahdieh Mehdipour, Hossein Mohammadi, Zohreh Mehdipour, Behzad Khorrami, Mahdi Nazari

**Affiliations:** 1 Department of Animal and Poultry Science, College of Aburaihan, University of Tehran, Tehran, Iran; 2 Department of Animal Science, College of Agriculture, University of Tabriz, Tabriz, Iran; 3 Department of Animal Science, Faculty of Agriculture and Natural Resources, Arak University, Arak, Iran; 4 Prestage Department of Poultry Science, North Carolina State University, Raleigh, NC 27606 USA

**Keywords:** Animal physiology, Reproductive biology

## Abstract

The aim of the present study was to investigate the effects of tempol and straw size on rooster sperm post-thaw quality and fertility. Rooster semen was cryopreserved in Lake extender containing 0 (control), 5, 10, 15 and 20 μM tempol (in two different straw size, 0.25 and 0.5). The percentage of total and progressive sperm motility, VAP and VSL increased in the 10 µM tempol group. Moreover, 10 µM tempol led to lower ROS compared to other groups. The lowest percentage of apoptotic-like changes was detected when the extender was treated with 10 µM of tempol. The minimum ROS was observed in the group treated with 0.5 straw size. Straw size did not have any significantly effect on GPx and SOD activities and TAC of frozen-thawed sperm. The highest significant percentage of fertility and hatching rate were observed in 10 µM of tempol. The results of the present study showed that supplementation of the Lake cryopreservation medium with 10 µM tempol improved cryo-survival. Also, the results of the present study suggested that Lake cryopreservation medium with 0.5-ml straw may perhaps be an appropriate method to improve the quality and fertility post-thawed rooster sperm.

## Introduction

Cryopreservation is a technology that allows living cells and tissues to be preserved for a long period of time at low cost. Optimal freezing media and cryoprotectant agent, on the other hand, has a significant effect on the success of sperm cryopreservation^1^. Changes in sperm membranes occur as a result of semen cryopreservation, resulting in cell damage or death. Furthermore, addition and removal of cryoprotectant in molar quantities may reduce the amount of live sperm after semen freezing and thawing. The plasma membrane of sperm is exposed to severe osmotic stress as a result, and its functional integrity is affected^2^. The principal cause of harm associated with sperm cryopreservation is oxidative stress. Oxidative stress is defined as a rise in the intracellular concentrations of reactive oxygen species (ROS)^3^. Sperm contain low levels of antioxidant enzymes (superoxide dismutase, glutathione peroxide, catalase, and glutathione reductase) and a high level of polyunsaturated fatty acids (PUFA)^4^.

Tempol (4-hydroxy-2,2,6,6-tetramethylpiperidine-1-oxyl), known as a member of nitroxide compounds family having superoxide dismutase mimetic activity, protects spermatozoa against oxidative stress, improves motility, viability and DNA integrity post thawing^5^. Tempol leads to the conversion of superoxide to less toxic oxidant, the hydrogen peroxide (H_2_O_2_). Researchers believe tempol can protect cells from intracellular ROS since it is a cell permeable antioxidant^6^. Mata-Campuzano et al.^7^ reported that tempol reduced ROS and DNA damage in the presence of oxidative stress. Moreover, adding 1-mM tempo or tempol to alpaca sperm at 10 °C increased motility and reduced DNA fragmentation after cryopreservation^8^.

In avian species, sperm freezing is an appropriate method of retaining sperm cells; nonetheless, thermal shock is an issue when cryopreserving avian sperm^9,10^. During the final stages of differentiation, spermatozoa lose an extensive part of their defensive antioxidants by losing part of its cytoplasm^11^. It is thought that addition of antioxidants to the extender before cryopreservation can partially compensate for this. A lot of factors influence the quality of sperm following cryopreservation, including the kind of sperm package^12^. 0.5 ml straws, for example, have a higher surface-to-volume ratio compared to 0.25 straws, allowing for more uniform freezing and thawing temperatures across the sample^13,14^. Ansari et al.^15^ observed packing sperm in 0.25 ml straws resulted in higher quality of sperm after freeze-thawing than 0.5 ml straws. Nöthling and Shuttleworth^16^, discovered that after 60 min post-thaw, 0.5-ml straws had 5.7 percent more progressively motile sperm and 6.5 percent less abnormal acrosomes than 0.25 straws, indicating that 0.5 ml straws are better for semen cryopreservation. These studies found that Equex-STM improves the quality of cryopreserved boar sperm, and that this cryopreservation procedure was suitable for a 0.5 ml straw.

Improving cryosurvival of spermatozoa by packing in various straws and employing antioxidants may enable frozen semen a feasible solution for preserving livestock and poultry genetics by increasing cryosurvival and subsequent fertility. Therefore, our objectives were to investigate Tempol and straw size and their effects on rooster sperm after freezing/thawing procedure.

## Materials and methods

All the chemicals used in the present study were prepared by Merck Company. A Lake-based medium was used to make the diluent. All animal care protocols were performed in accordance with ARRIVE guidelines and the University of Tehran guidelines for Animal Experiments. The Animal Research Committee of the University of Tehran authorized the animal study (IR. REC.1396.579).

### Animals used

In this experiment, 10 Ross roosters aged 30 weeks were used. The roosters were kept in individual cages in the research hall of the University of Tehran, using a timer with an exposure program of 15 h of light and 9 h of darkness. The room temperature was set in the range of 18 to 22 °C. All the roosters were fed a diet based on the Ross catalog.

### Sperm collection

Roosters were accustomed to sperm collection for 2 weeks. Semen samples were usually collected twice a week using abdominal massage method. Immediately after semen collection, sperm samples were transferred to a laboratory at 37 °C and initial assessment was done. The semen collected from the roosters was mixed together to remove individual effects. In this step, the volume of pooled semen was extended at 37 °C with Lake extender containing 0 (control), 5, 10, 15 and 20 μM tempol. After diluting the semen, a gradual cooling step was performed in a 4 °C refrigerator for 3 h. Then, the samples were pulled into 0.25 ml or 0. 5 ml freezing tubes. The samples were placed horizontally at nitrogen vapor for 7 min. After 7 min, they were immersed in liquid nitrogen. Finally, the straws were transferred to a nitrogen tank (− 196 °C) and kept at this temperature until the experiment.

### Computer sperm analyzer system (CASA)

The first parameters evaluated after freezing–thawing were sperm motility parameters. Post-thawed motion characteristics of sperm were analyzed by computer-assisted sperm motility analysis (CASA; IVOS version 12; HamiltonThorne Biosciences, MA) with settings programmed to detect rooster sperm (30 frames acquired, minimum contrast = 50, minimum average path velocity (VAP) = 5 mm/s, minimum straight line velocity (VSL) = 6 mm/s, non-motile head size = 7 pix and nonmotile head intensity = 95). At least 10 fields were randomly selected from each sample and the motility parameters of 300 sperm were analyzed by CASA system.

### Plasma membrane integrity test (HOST)

This test was used to evaluate the plasma membrane integrity of sperm cells^17^. To perform this test, 10 µl of the semen sample was mixed with 100 μl of hyposmotic solution and incubated for 30 min at 37 °C. Then, 5 μl of the mixed sample was placed on a preheated slide and covered with a cover slide. Then, 200 sperm were counted with a magnification of 400x, with swollen tail considered as sperm with healthy plasma membranes.

### Sperm morphology test

To evaluate the morphology of sperm, 10 μl of each semen sample was added to microtubes containing 150 μl of Hancock solution^18^. Then 5 μl of this mixture was placed on a slide and covered with a slide and at least 200 sperm were counted under a phase contrast microscope with a magnification × 400.

### Assessing viability and apoptotic-like changes

Sperm cell viability and apoptotic-like changes were assessed using the Annexin V-FITC (IQP-116F, PSD Kit, the Netherland) programmed cell death assay^10^. The procedure contained thawing the semen samples and removing the diluent by centrifugation. Then the samples were washed with calcium buffer and 10 μl of annexin-V was added to the semen samples and the samples were stored in a dark place for 20 min. After 20 min, 10 μl of PI was added to the sample. Then, the rate of phosphatidylserine transfer of sperm membrane in the samples was evaluated by flow cytometer (FACSCalibur, Becton Dickinson, San Jose, CA, USA). In the diagram provided by the flow cytometer, the samples with annexin-V negative and PI negative (A−/PI−) and with annexin-V positive and PI negative (A+/PI−) were considered as viable sperm and alive but primary apoptosis, respectively. The samples with annexin-V positive and PI positive (A+/PI+), were considered as dead sperm.

### Evaluation of mitochondrial activity using Rhodamine-123

To perform this test, 10 μl of rhodamine was added to the sample and placed in a dark place at room temperature for 20 min. After 20 min, 10 µl of PI were added to the sample. The mitochondrial activity of the samples was then measured by flow cytometry. In the diagram given by the flow cytometer, if the sample is rhodamine positive and PI negative (PI−/R123+), it is considered as active mitochondria, and if the rhodamine is positive and PI positive (R123+/PI+), it is considered as inactive mitochondria^10^.

### ROS measurement

Measurements of ROS were performed using the method of Mehdipour et al.^17^. Briefly, the semen samples were incubated for 20 min at 37 °C in 250 µl of PBS. The samples were centrifuged for 7 min at 300×*g*, and the supernatant was extracted. After centrifuging the pellet at 300×*g* for 7 min, 3 mL of PBS was added to the pellet. By diluting the sperm with PBS, the concentration was adjusted to 20 × 10^6^ ml^−1^. The tubes containing 400 µl of sample were then filled with ten microliters of luminol (5 mM, 5-amino-2,3-dihydro-1,4-phthalazinedione; Sigma Chemical, St. Louis, MO) and put in an Orion II Microplate Luminometer (Berthold Detection Systems Gmbh, Germany). 10^3^ counted photons per minute (cpm) per 10^6^ spermatozoa were used to measure ROS.

### Total antioxidant capacity test

Measurements of total antioxidant capacity were performed using the TEAC Randox kit method (RANDOX Laboratories Ltd.). The TEAC method is based on inhibition with ABTS cation radical scavenger antioxidants. 20 μl of the samples were mixed with 1 ml of chromogen (ABTS reagent) and the reaction was initiated by adding 200 μl of H_2_O_2_. In this method, a stable blue-green color with a maximum light absorption of 600 nm is produced, which can be measured by spectrophotometry.

### Evaluation of glutathione peroxidase activity

GPX enzyme activity was measured using Ransox kit (RANDOX Laboratories Ltd.). 10 μl of sample was mixed with 500 µl of GPx Reagent (Glutathione, Glutathione Reductase and NADPH) and 10 µl of Buffer (EDTA and Phosphate Buffer), then the suspension was mixed with 4 µl cumene hydroperoxide. Next, the absorption was measured at a wavelength of 340 nm.

### Evaluation of superoxide dismutase activity

Measurement of superoxide dismutase (SOD) activity was performed using spectrophotometry (RANDOX Laboratories Ltd.). 50 μl of the samples were mixed with 1.7 mL of mixed substrate (xanthine and Int) and then the solution was mixed with 500 μl Xanthine Oxidase. Finally, the absorption was measured at a wavelength of 505 nm.

### Fertility, and hatchability

We employed artificial insemination for the best group of tempol (10 µM) and both straw sizes based on the findings of in-vitro sperm parameters to assess fertility and compared them with the control group (without any antioxidant and both straw sizes). Artificial insemination (AI) was carried out according to the technique of Long and Kulkarni^19^ with a minor modification. Breeder hens (Ross) were divided into 4 treatments (30 hens in each treatment). Next, 4 treatments were selected according to our results of in vitro sperm determination. Glycerol was removed using a discontinuous Accudenz gradient, which contained a 30% (0.5 ml) layer under a 12% (5.0 ml) layer. Semen was centrifugated (1200 rcf; 20 min), consequently, the glycerol and extender placed above the 12% layer, and the sperm cells were deposited between the 12 and 30% layers. Then, the upper layer was removed and the 30% layer was aspirated. After Accudenz, up to 500 µl extender was added to the semen layer. Artificial insemination was performed after thawing at 15 pm on certain days (twice a week) with insemination of 100 × 10^6^ sperm/straw obtained from each treatment. Up to 5 days after the final AI, the eggs were collected. The eggs were incubated in the setter for 18 days before being transferred to the hatchery for the final three days. After 7 days of incubation, the fertility rate (by candling of eggs) [(embryonated eggs/total eggs) × 100], and after 21 days hatchability of fertile eggs [(hatched chick numbers/fertilized) × 100] were evaluated.

### Statistical analysis

Eight replicates of pooled semen were used for cryopreservation and evaluation of sperm occurred as previously described in this manuscript. All data were analyzed for normal distribution by PROC UNIVARIATE and the Shapiro–Wilk test. The Mixed procedure of SAS version 9.1 was used to examine the data (SAS Institute, 2002, Cary, NC). The main effects (tempol and straw size) and their interactions were included in the mathematical model. Each component was considered as a main effect because the model revealed no significant interaction between tempol and straw size. Tukey's test was used to evaluate statistical differences between the treatment group means. Differences with a P value of less than 0.05 were regarded statistically significant. The findings were presented in terms of least squares means and standard error of the mean.

## Results

### Motility and velocity parameters

Effects of different levels of tempol on the motility parameters of post-thawed sperm are presented in Table 1. The percentage of total and progressive sperm motility increased in group of 10 µM tempol (68.7 ± 1.2 and 29.1 ± 0.7) compared to the control group (41.8 ± 1.2 and 18.0 ± 0.7, P < 0.05). Significantly higher percentages (P < 0.05) of VAP, VSL and LIN were observed in the samples treated with 10 µM tempol (36.2 ± 0.65, 22.2 ± 0.6 and 36.3 ± 1.3, respectively) compared to other groups. For the values of the VCL, STR, ALH and BCF variables, there were no differences (P > 0.05) after addition of tempol at different levels.

**Table 1:** Effect of different levels of tempol on motility parameters of rooster sperm.

Antioxidant (µM)	TM (%)	PM (%)	VAP (µm/s)	VSL (µm/s)	VCL (µm/s)	LIN (%)	STR (%)	ALH (µm)	BCF (Hz)
0	41.8^c^	18.0^c^	31.4^b^	17.2^b^	55.5	31.3^b^	55.0	5.2	16.4
5	53.1^b^	22.3^b^	32.7^b^	18.0^b^	56.8	31.9^b^	55.8	4.8	16.7
10	68.7^a^	29.1^a^	36.2^a^	21.2^a^	58.7	36.3^a^	58.8	4.4	17.6
15	50.2^b^	21.4^b^	32.4^b^	17.6^b^	56.0	31.6^b^	54.4	4.9	16.6
20	40.3^c^	17.5^c^	31.4^b^	17.2^b^	55.3	31.3^b^	55.1	5.3	16.4
SEM	1.2	0.7	0.65	0.6	1.1	1.3	2.1	0.2	0.5
P- value	< 0.0001	< 0.0001	< 0.0001	< 0.0001	0.229	0.028	0.637	0.069	0.595

Different superscripts within the same column indicate differences among groups (P < 0.05).

*TM* total motility, *PM* progressive motility, *VSL* straight–line velocity, *VAP* average path velocity, *VCL* curvilinear velocity, *LIN* linearity, *STR* straightness, *ALH* mean amplitude of the lateral head displacement, *BCF* mean of the beat cross frequency.

### Plasma membrane functionality, sperm abnormalities and mitochondria activity

**Figure 1:**
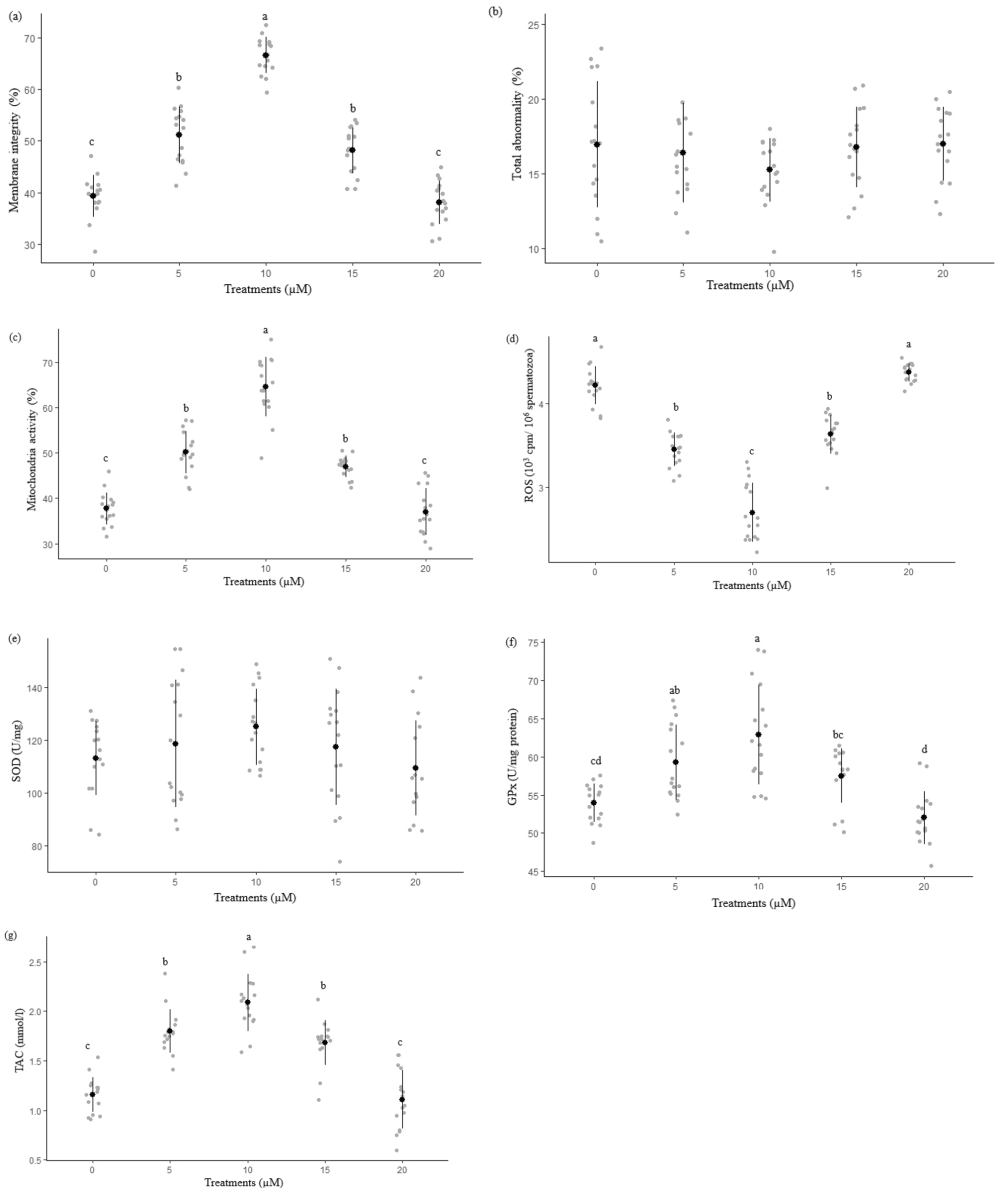
Membrane integrity (**a**), Total abnormality (**b**), mitochondrial activity (**c**), ROS (**d**), SOD (**e**), GPx (**f**) and TAC (**g**) after thawing samples cryopreserved with the different tempol concentrations. Different letters indicate that treatments differ P < 0.05.

The highest percentages of plasma membrane functionality, and mitochondria activity were found in the 10 µM tempol (66.6 ± 0.9 and 64.5 ± 1.1, respectively) compared to other treatments (Fig. 1a,c) (P < 0.05). Extender supplementation with different levels of tempol during cryopreservation had no significant effect (P > 0.05) on rooster cryopreserved sperm abnormal morphology rate (P > 0.05) (Fig. 1b).

### Viability and apoptotic-like changes

The results of viability and apoptotic-like changes in rooster sperm cryopreservation are depicted in Table 2. The percentage of viable sperm was the highest when storage was in the extender treated with 10 µM tempol (65.8 ± 1.1) compared to all treatments (P < 0.05). The lowest percentage (P < 0.05) of and apoptotic-like changes sperm was discovered when the extender treated with 10 µM tempol (15.7 ± 0.9) (P < 0.05). The sperm stored in the extender treated with 10 µM tempol (18.4 ± 1.4) had the lowest (P < 0.05) dead sperm compared to other groups.

**Table 2:** Effect of different levels of tempol on viability and apoptotic-like changes of rooster sperm after freeze-thawing.

Antioxidant (µM)	Viable sperm (%)	Apoptotic sperm (%)	Dead sperm (%)
0	38.1^c^	24.1^ab^	37.6^a^
5	49.2^b^	20.6^b^	30.0^b^
10	65.8^a^	15.7^c^	18.4^c^
15	46.5^b^	21.8^ab^	31.5^b^
20	37.5^c^	24.5^ab^	37.9^a^
SEM	1.1	0.9	1.4
P- value	< 0.0001	< 0.0001	< 0.0001

Different letters within the same columns show significant differences among the groups (P < 0.05).

### Biochemical parameters

Figure 1d–g indicate the data related to ROS, superoxide dismutase (SOD) and glutathione peroxidase (GPx) activities and total antioxidant capacity (TAC), respectively. In case of ROS, 10 µM tempol (2.7 ± 0.05) illustrated lower ROS compared to other groups (P < 0.05). No significant difference (P > 0.05) was detected among groups in case of SOD. For GPx and TAC, 10 µM tempol (62.8 ± 1.1 and 2.0 ± 0.06, respectively) presented the highest GPx and TAC among groups.

### Effect of straw size on sperm parameters

The effect of straw size on motile parameters after cryopreservation are displayed in Table 3. Total and progressive motility were significantly higher in 0.5 straw size (52.2 ± 0.7 and 22.7 ± 0.4, respectively) compared to 0.25 straw size (P < 0.05). Straw size did not have any significant effects on VAP, VSL, LIN, VCL, STR, ALH and BCF (P > 0.05). The membrane integrity and mitochondria activity of sperm cryopreserved with 0.5 straw size (50.6 ± 0.6 and 48.9 ± 0.7, respectively) had better performance compared to 0.25 straw size (Table 4) (P < 0.05). Based on the data shown in Table 5, the minimum ROS is observed in the group of 0.5 straw size (3.5 ± 0.03) (P < 0.05). But straw size did not have any significant effects on the GPx and SOD activities and TAC of frozen-thawed sperm (P > 0.05). Effect of straw size on viable, and apoptotic-like changes, and dead of rooster sperm after cryopreservation are presented in Table 6. When comparing 0.5 straw size (49.5 ± 0.7) to 0.25 straw size, the percentage of viable sperm was considerably higher (P < 0.05). In comparison to the 0.25 straw size group, the 0.5 straw size group (29.6 ± 0.9) had the lowest percentage of dead sperm (P < 0.05). Moreover, straw size did not have any significant effects on apoptotic-like changes frozen-thawed sperm among groups (P > 0.05).

**Table 3:** Effect of straw size on motile parameters of rooster sperm.

Motility parameters	Straw size		SEM	P- value
	0.25	0.5		
TM (%)	49.5^b^	52.2^a^	0.7	0.013
PM (%)	20.7^b^	22.7^a^	0.4	0.003
VAP (µm/s)	32.3	33.4	0.4	0.054
VSL (µm/s)	17.9	18.6	0.3	0.147
VCL (µm/s)	56.1	56.8	0.7	0.519
LIN (%)	32.0	33.0	0.8	0.415
STR (%)	55.6	56.0	1.3	0.839
ALH (µm)	5.1	4.8	0.15	0.253
BCF (Hz)	16.5	16.9	0.3	0.406

Different superscripts within the same row indicate differences among groups (P < 0.05).

*TM* total motility, *PM* progressive motility, *VSL* straight–line velocity, *VAP* average path velocity, *VCL* curvilinear velocity, *LIN* linearity, *STR* straightness, *ALH* mean amplitude of the lateral head displacement, *BCF* mean of the beat cross frequency.

**Table 4:** Effect of straw size on plasma membrane integrity, total abnormalities and mitochondria activity thawed rooster sperm.

Parameters	Straw size		SEM	P- value
	0.25	0.5		
Membrane integrity (%)	46.7^b^	50.6^a^	0.6	< 0.0001
Total abnormality (%)	16.6	16.3	0.5	0.646
Mitochondria activity (%)	45.6^b^	48.9^a^	0.7	0.001

Different letters within the same row show significant differences among the groups (P < 0.05).

**Table 5:** Effect of straw size on ROS, glutathione peroxidase (GPx) and superoxide dismutase (SOD) activities and total antioxidant capacity (TAC) of rooster thawed semen.

Parameters	Straw size		SEM	P-value
	0.25	0.5		
ROS (10^3^ cpm/10^6^ sperm)	3.7^a^	3.5^b^	0.03	< 0.0001
SOD (U/mg)	115.1	118.3	3.7	0.460
TAC (mmol/l)	1.5	1.6	0.04	0.218
GPx (U/mg protein)	56.4	57.7	0.7	0.200

Different superscripts letters within row are significantly different (P < 0.05).

**Table 6:** Effect of straw size on viability and apoptotic-like changes and dead of thawed rooster sperm.

Parameters	Straw size		SEM	P-value
	0.25	0.5		
Viable (%)	45.3^b^	49.5^a^	0.7	< 0.0001
Apoptotic (%)	21.9	20.8	0.5	0.163
Dead (%)	32.6^a^	29.6^b^	0.9	0.020

Different superscripts letters within row are significantly different (P < 0.05).

### Reproductive performance

Fertility and hatching rate were higher (P < 0.05) in 10 µM tempol with 0.25 straw size (60.5 and 72.7, respectively) and 10 µM tempol with 0.5 straw size (64 and 74.2, respectively) compared to 0 µM tempol with 0.25 straw size and 0 µM of tempol with 0.5 straw size (Table 7) (P < 0.05).

**Table 7:** Effect of tempol and straw size on fertility and hatchability rates of rooster semen after freeze-thawing.

Treatments	Parameters		
	Fertilized eggs	Hatched eggs	Hatched eggs ratio (hatched/fertilized, %)
0 µM of tempol and 0.25 straw size	80 (40)^b^	52 (26)^b^	65^b^
10 µM of tempol and 0.25 straw size	121 (60.5)^a^	88 (44)^a^	72.7^a^
0 µM of tempol and 0.5 straw size	85 (42.5)^b^	56 (28)^b^	65.8^b^
10 µM of tempol and 0.25 straw size	128 (64)^a^	95 (47.5)^a^	74.2^a^

Each experimental group contained 200 eggs initially. Numbers are absolute counts of eggs, with percentages (ratio respect to the initial egg count) between parentheses, except for the hatched eggs ratio.

Different superscripts letters within row are significantly different (P < 0.05).

## Discussion

Cryopreservation impairs the antioxidant defensive system (such as GPx, SOD and catalase) and has deleterious effects on rooster sperm quality, which decreases the survival and fertility capacity of thawed sperm^20^. The results of various studies have shown that supplementing sperm medium with antioxidants reduces the impact of ROS-induced damage on sperm during freezing thawing^20,21^. In the present study, we evaluated the different levels of tempol and straw size for improving sperm cryotolerance and preventing the loss of quality and fertility capacity.

Our findings revealed that sperm treated with 10 μM tempol had considerably higher motility and viability comparing to control. The current investigation found that tempol had some favorable effects on motility and velocity parameters including TM and PM as well as VAP, VSL and LIN that is in line with a study in which tempol significantly improved motility and viability post thawing^22^. Moreover, in a study on cryopreservation of alpaca sperm, the authors showed that loss of motility during cryopreservation could be partially prevented by supplementation of semen extender with 1 mM tempol^23^ which approves the motility results of the present study. Likewise, these findings support another study showing that supplementation of human sperm with 5 µM tempol increased sperm motility and viability after freezing^5^. However, in contrast to our findings, Mata-Campuzano et al.^7^ found that tempol decreased sperm motility. Also, in contrary to our result, Foote et al.^24^, indicated that tempol produced harmful effects on bull spermatozoa frozen in whole milk extender. The variation in the results may be due to the differences in laboratory techniques, species, freezing procedure, type of extender, photoperiod program, age of animals, diet formulations and the antioxidant levels.

Increasing cryosurvival of rooster spermatozoa by packaging them in different size of straws, using different diluents, or freezing sperm at different concentrations, as well as using alternative cryoprotectants, may make frozen semen a viable option for preserving poultry genetics. Since 0.5 ml straws have higher surface-to-volume ratio than 0.25 ml straws, sperm freezing should be faster in the 0.25 ml straws. As a result, we expected that post-thaw quality of rooster sperm frozen in 0.25 and 0.5 ml straws using the same cryopreservation procedure (positioned 4 cm above LN2 for 7 min) would differ. Nevertheless, sperm inserted into both types of straws had equal fertilizing potential. In contrary to our results, animals in other studies (dogs and boars) had better sperm quality frozen in 0.5 ml straws compared with 0.25 ml straws^16,25^. Buranaamnuay et al.^25^, found that 0.5-ml straws had greater percentages of viable sperm than 0.25-ml straws which was in line with our results.

Plasma membrane integrity during the freeze–thaw process has been shown to be connected with post-thaw viability, motility, and fertilization capacity^11^. Various treatments had no effect on the morphology of sperm in this investigation. Our findings support prior research that showed antioxidant supplementation did not affect the sperm morphology^9,10^. In the current study, addition of tempol to the semen extender increased the number of living sperm while decreasing the number of apoptotic-like changes and dead sperm. These beneficial effects can be attributed to the critical function that tempol plays in enhancing antioxidant capacity, which maintains the sperm plasma membrane from reactive oxygen species (ROS)^5^. ROS are highly reactive chemicals formed from O_2_ which causes DNA damage, sperm death, and low sperm motility^26^. It would be imperative to define antioxidant concentration that can reduce ROS production to the level required for maintaining sperm motility without having deleterious effects. However, it is difficult to define such concentration and requires substantial manipulation and the defined concentration may vary between different semen samples. It was interesting to note that addition of 10 µM Tempol significantly reduced the ROS production due to its potential to decrease the generation of highly toxic hydroxyl radicals in Fenton or Haber–Weiss reactions mediated with free iron or cupper.

The advantageous effects of tempol can be attributed to its cell permeability characteristic and ability to reduce superoxide production both at intra- and extra-cellular levels^22^. Tempol has been considered to be a cell-penetrating antioxidant, which scavenges superoxide anions and acts as an SOD mimetic agent. It promotes the transformation of O^−^_2_ to H_2_O_2_ and decreases the generation of hydroxyl radicals, which have been identified as the most toxic agents^22^. Santiani et al.^27^ studied the effects of tempol on ram sperm cryopreservation and concluded that tempol as a superoxide inducing agent, blocks the hydroxyl radical formation in ram spermatozoa because of reduction in SOD activity after freezing which is in contrast to our study. Using higher concentrations of tempol may be harmful to sperm cells as excessive addition of tempol at 20 µM showed lower quality parameters in the present study. It has been stated that high concentrations of antioxidants can change plasma membrane fluidity and make sperm more susceptible to lipid peroxidation^28^. Therefore, choosing the most appropriate level of antioxidant is crucial for sperm quality after freeze-thawing.

During cryopreservation, sperm are subjected to freezing shocks, which results in a reduction in their quality after they have been thawed^9^. As a result, adding antioxidants to the freezing media may increase sperm viability and, consequently, make sperm transit through the female avian reproductive tract easier. Increased sperm integrity during transit through the reproductive tract is a result of optimal antioxidant supplementation^17^. In the present study, supplementation with 10 µM tempol resulted in improved fertility and hatchability of incubated eggs. In agreement to our results, in a study on ram semen diluted with media containing tempol, an increase in fertility was observed^29^. Also in other studies, using tempol in combination with liquid semen held in cooling conditions led to high motility rates and good fertility in goat^30^, and ram^31^. Sperm motility has a direct correlation with fertilizing ability^32^. In avian species, motility (particularly progressive motility) has the greatest influence on extending the fertilization time^33^. Because inseminated dosages are kept in sperm storage tubules (SSTs) for a period of time before being sent to the fertilization site, spermatozoa motility is particularly important in avian species^34^.

## Conclusion

The results of the present study showed that supplementation of the Lake cryopreservation medium with 10 µM tempol improved cryo-survival and preservation of sperm motility, membrane functionality, mitochondrial activity and viability. Additionally, using 10 µM tempol resulted in lower apoptosis and lipid peroxidation of rooster sperm cells after thawing. The results indicated that tempol might be considered as a novel potential cryoprotectant for the storage of rooster sperm in freezing media. The results of this investigation suggested that improved post-thawed rooster sperm quality, led to superior freeze/thaw characteristics, which may be achieved by using Lake cryopreservation mixture with 0.5-ml straw. Tempol and 0.5 ml straw could be beneficial for freezing rooster semen, especially for improving specific protocols (Supplementary Information).

## Supplementary Information

Supplementary Information.

## Data Availability

The authors declare that the data supporting the findings of this study are available within the paper.
